# Supplementary material for: Inclusion of initial caries lesions in a population-based sample of Brazilian preschool children: Impact on estimates and treatment needs
Source: PLoS One. 2020 Jun 19;15(6):e0234122. doi: 10.1371/journal.pone.0234122 (PMC7304588; doi:10.1371/journal.pone.0234122)
Supplement: Attachment — Submitted filename: PONE-D-19-32314_reviewer plos one.pdf [file pone.0234122.s006.pdf]

PLOS ONE

Inclusion of initial caries lesions in a population-based sample of Brazilian preschool children: impact on estimates and treatment needs

--Manuscript Draft--

**Manuscript Number:**	PONE-D-19-32314
**Article Type:**	Research Article
**Full Title:**	Inclusion of initial caries lesions in a population-based sample of Brazilian preschool children: impact on estimates and treatment needs
**Short Title:**	Initial caries lesions: impact and surveys
**Corresponding Author:**	Patrícia Carvalho, M.D<br>Universidade de Sao Paulo<br>Sao paulo, BRAZIL
**Keywords:**	dental caries; initial caries lesions, primary teeth, preschool, management
**Abstract:**	The aim of this study was toassess changes in epidemiological estimates and treatment needed according to ICCMSwhenincluding initial caries lesions in a population-based sample of preschool children. A cross-sectional survey was conducted in a Brazilian municipality, collecting data of preschool children in 16 health centers. Caries detection used the merged codes (epi-codes) for ICDAS/ICCMS. An option for treatment according to ICCMS was chosen during examination. Caries experience (dmft/dmfs) and prevalence were estimated considering 3 thresholds (A- initial, moderate and severe lesions, B- only moderate and severe lesions and C- severe caries lesions). Incremental need for non-operative care was also verified. The sample consisted of 663 children aged 2-4 years (response rate 99.85%). Including initial lesions, a 2-fold increase in dmft was observed (A: 3.36, B: 1.02, p<0.001). With the inclusion, the caries prevalence was increased in 75% compared to threshold B (28%). From 56% of children who required any intervention, most of them (76%) should be essentially scheduled for non-operative care. It can be concluded that including initial caries lesions in an epidemiological survey may significantly impact on population caries experience assessment.
**Order of Authors:**	Patrícia Carvalho, M.D
	Gustavo Tello
	Jenny Abanto
	Luciana Oliveira
	Marcelo Bönecker
	Mariana Braga
**Additional Information:**	
**Question**	**Response**
**Financial Disclosure**<br><br>Enter a financial disclosure statement that describes the sources of funding for the work included in this submission. Review the submission guidelines for detailed requirements. View published research articles from *PLOS ONE* for specific examples.<br><br>This statement is required for submission and **will appear in the published article** if	The author(s) received no specific funding for this work.

the submission is accepted. Please make sure it is accurate.<br><br>**Unfunded studies**<br>Enter: *The author(s) received no specific funding for this work.*<br><br>**Funded studies**<br>Enter a statement with the following details:<br>• Initials of the authors who received each award<br>• Grant numbers awarded to each author<br>• The full name of each funder<br>• URL of each funder website<br>• Did the sponsors or funders play any role in the study design, data collection and analysis, decision to publish, or preparation of the manuscript?<br>• **NO** - Include this sentence at the end of your statement: *The funders had no role in study design, data collection and analysis, decision to publish, or preparation of the manuscript.*<br>• **YES** - Specify the role(s) played.<br><br>* typeset	
**Competing Interests**<br><br>Use the instructions below to enter a competing interest statement for this submission. On behalf of all authors, disclose any competing interests that could be perceived to bias this work—acknowledging all financial support and any other relevant financial or non-financial competing interests.<br><br>This statement **will appear in the published article** if the submission is accepted. Please make sure it is accurate. View published research articles from *PLOS ONE* for specific examples.	The authors have declared that no competing interests exist.

**NO authors have competing interests**<br><br>Enter: *The authors have declared that no competing interests exist.*<br><br>**Authors with competing interests**<br><br>Enter competing interest details beginning with this statement:<br><br>*I have read the journal's policy and the authors of this manuscript have the following competing interests: [insert competing interests here]*<br><br>* typeset	
**Ethics Statement**<br><br>Enter an ethics statement for this submission. This statement is required if the study involved:<br><br>• Human participants<br>• Human specimens or tissue<br>• Vertebrate animals or cephalopods<br>• Vertebrate embryos or tissues<br>• Field research<br><br>Write "N/A" if the submission does not require an ethics statement.<br><br>General guidance is provided below. Consult the submission guidelines for detailed instructions. **Make sure that all information entered here is included in the Methods section of the manuscript.**	The study has been approved by the Research Ethics Committee, School of Dentistry, University of São Paulo by<br>CAAE: 47209015.4.0000.0075 and Opinion Number: 1.167.931

Powered by Editorial Manager® and ProduXion Manager® from Aries Systems Corporation

**Format for specific study types**<br><br>**Human Subject Research (involving human participants and/or tissue)**<br>• Give the name of the institutional review board or ethics committee that approved the study<br>• Include the approval number and/or a statement indicating approval of this research<br>• Indicate the form of consent obtained (written/oral) or the reason that consent was not obtained (e.g. the data were analyzed anonymously)<br><br>**Animal Research (involving vertebrate animals, embryos or tissues)**<br>• Provide the name of the Institutional Animal Care and Use Committee (IACUC) or other relevant ethics board that reviewed the study protocol, and indicate whether they approved this research or granted a formal waiver of ethical approval<br>• Include an approval number if one was obtained<br>• If the study involved *non-human primates*, add *additional details* about animal welfare and steps taken to ameliorate suffering<br>• If anesthesia, euthanasia, or any kind of animal sacrifice is part of the study, include briefly which substances and/or methods were applied<br><br>**Field Research**<br><br>Include the following details if this study involves the collection of plant, animal, or other materials from a natural setting:<br>• Field permit number<br>• Name of the institution or relevant body that granted permission	
**Data Availability**<br><br>Authors are required to make all data underlying the findings described fully available, without restriction, and from the time of publication. PLOS allows rare exceptions to address legal and ethical concerns. See the PLOS Data Policy and FAQ for detailed information.	Yes - all data are fully available without restriction

A Data Availability Statement describing where the data can be found is required at submission. Your answers to this question constitute the Data Availability Statement and **will be published in the article**, if accepted.<br><br>**Important:** Stating 'data available on request from the author' is not sufficient. If your data are only available upon request, select 'No' for the first question and explain your exceptional situation in the text box.<br><br>Do the authors confirm that all data underlying the findings described in their manuscript are fully available without restriction?	
**Describe where the data may be found in full sentences. If you are copying our sample text, replace any instances of XXX with the appropriate details.**<br><br>• If the data are **held or will be held in a public repository**, include URLs, accession numbers or DOIs. If this information will only be available after acceptance, indicate this by ticking the box below. For example: *All XXX files are available from the XXX database (accession number(s) XXX, XXX.*).<br>• If the data are all contained **within the manuscript and/or Supporting Information files**, enter the following: *All relevant data are within the manuscript and its Supporting Information files.*<br>• If neither of these applies but you are able to provide **details of access elsewhere**, with or without limitations, please do so. For example:<br><br>*Data cannot be shared publicly because of [XXX]. Data are available from the XXX Institutional Data Access / Ethics Committee (contact via XXX) for researchers who meet the criteria for access to confidential data.*<br><br>*The data underlying the results presented in the study are available from (include the name of the third party*	If this information will only be available after acceptance in Supporting Informational Files.

and contact information or URL).<br>• This text is appropriate if the data are owned by a third party and authors do not have permission to share the data.<br><br>* typeset	
Additional data availability information:	Tick here if the URLs/accession numbers/DOIs will be available only after acceptance of the manuscript for publication so that we can ensure their inclusion before publication.

Powered by Editorial Manager® and ProduXion Manager® from Aries Systems Corporation

Manuscript Click here to download Manuscript Manuscript .docx

PLOS | ONE
TITLE, AUTHOR, AFFILIATIONS FORMATTING GUIDELINES

Inclusion of initial caries lesions in a population-based sample of Brazilian preschool children: impact on estimates and treatment needs

Initial caries lesions: impact and surveys

Patrícia de Carvalho[1*¶], Gustavo Tello[2&], Jenny Abanto[1&], Luciana Butini Oliveira[3&], Marcelo Bönecker[1&], Mariana Minatel Braga[1¶].

[1]Department of Orthodontics and Pediatric Dentistry, Dental School, University of São Paulo-USP, São Paulo, Brazil,

[2]Division of Biomedical Sciences, Department of Clinics of the University Center of Los Altos, University of Guadalajara, Tepatitlán, Jalisco, Mexico and

[3]Division of Pediatric Dentistry, Faculdade São Leopoldo Mandic, Campinas, Brazil.

* Corresponding author

E-mail: patcarvalho@usp.br (PC)

[¶]These authors contributed equally to this work.

[&]These authors also contributed equally to this work.

Abstract

The aim of this study was to assess changes in epidemiological estimates and treatment needed according to ICCMS when including initial caries lesions in a population-based sample of preschool children. A cross-sectional survey was conducted in a Brazilian municipality, collecting data of preschool children in 16 health centers. Caries detection used the merged codes (epi-codes) for ICDAS/ICCMS. An option for treatment according to ICCMS was chosen during examination. Caries experience (dmft/dmfs) and prevalence were estimated considering 3 thresholds (A- initial, moderate and severe lesions, B- only moderate and severe lesions and C- severe caries lesions). Incremental need for non-operative care was also verified. The sample consisted of 663 children aged 2-4 years (response rate 99.85%). Including initial lesions, a 2-fold increase in dmft was observed (A: 3.36, B: 1.02, $p<0.001$). With the inclusion, the caries prevalence was increased in 75% compared to threshold B (28%). From 56% of children who required any intervention, most of them (76%) should be essentially scheduled for non-operative care. It can be concluded that including initial caries lesions in an epidemiological survey may significantly impact on population caries experience assessment.

Introduction

National epidemiological surveys to evaluate oral health conditions in children and guide plans in public oral care and public health strategies are important. In Brazil, this kind of surveys has been carried out since the 1980s. There has been a trend towards a decrease in dental caries indicators, mainly in 12-year-old children, but the same is not evident in the 5-year group.[1] The absence of priority of this age group in the agenda of public health services could be a possible explanation. Therefore, understanding what may be occurring differentially in this population is necessary to guide public health policies.

It has been advocated that noncavitated caries, if early detected, could be arrested through preventive management and restorative treatment could be avoided, hence preserving the dental structure and lowering the costs of treatment.[2,3,4] Due to that, the inclusion of these lesions in epidemiological surveys could offer us a screenshot of the demand for this type of care. On the other hand, the national epidemiological surveys do not assess the initial caries lesions. Due to the high number of cavitated caries lesions in Brazil, it is understandable that initial caries lesions are not the priority to guide dental care in public health. On the other hand, if looking for a deeper understanding or mapping about the severity of the disease among children, including initial lesions could be an interesting choice.

The inclusion of initial caries in epidemiological surveys is possible using ICDAS[5,6], but it is necessary additional time for the examination and it can result in a decrease in examiner reliability at noncavitated thresholds.[5] The use of merged ICDAS codes could be used in epidemiological surveys.[10,11] On the other hand, it could

simplify the calibration process for epidemiological purposes while guarantee the mapping of caries lesions in all severity stages.

Recently, The International Caries Classification and Management System (ICCMS™) integrating the ICDAS™ as caries process staging to the management of lesions was proposed.[7] This system is flexible to merge ICDAS scores since some scores present similar management.[8,9] Regarding decision-making, the ICCMS advocates the prevention of new caries lesions and on stopping the progression of existing lesions. Thus, it preserves the structure of tooth with Non-Operative Care of lesions in early stages and Tooth Preserving Operative Care of severe lesions.[7] In addition, it could allow to guide appropriate public policy for diagnosis.

Therefore, the aim of this study was to assess changes in epidemiological estimates and treatment needs when including initial caries lesions in a pre-school children sample. For this purpose, ICDAS merged codes and ICCMS clinical decision making tree was used. This is the first study to show this impact in a population-based survey.

Material and Methods

This manuscript was reported accordingly the recommendations of the STROBE Statement - checklist of items that should be included in reports of observational studies.[12]

Ethical Considerations

The study has been approved by the Research Ethics Committee, School of Dentistry, University of São Paulo. All parents/caregivers received information regarding the aim of this study and signed informed consent forms.

Study population and data collection

A descriptive cross-sectional study was undertaken in children from 2 to 4 years old, living in Maua, São Paulo, Brazil. Maua had a population of 417,064 inhabitants in 2010, including 23,082 children under 5 years old. The per capita income was around US$ 126.24/month and Human Development Index of 0.781.[13] The city has a fluoridated water supply (0.7ppm).

As this is the epidemiological survey in the municipality of Maua, the prevalence of dental caries among 5-year-old children was based on a previous national survey.[14] The sample size was calculated to give a standard error of 5%, a design effect of 1.4 with a 95% confidence interval. To cover non-response, the sample was increased by 20% totalizing 642 children.

Participants were systematically selected from all children attending each of the 16 health centers in 2015 during the National Children`s Vaccination Day in Maua, Sao Paulo.

Children were equally selected in 16 health centers in municipality. Each fifth child in the vaccination queue was invited to participate. If parents/caregivers did not agree to participate or child who showed signs of non-cooperative conduct, the next child in the queue was selected. To avoid possible biases, relatives and children living in the same household as the selected child were excluded from the study. Also, only children whose parents were present were included in the sample, to ensure that the questionnaire was answered. Children with systemic and/or neurological diseases and insufficient data were excluded of sample. This methodology was used in previous surveys carried out by the same authors.[15-20]

The sample presented a prevalence of 4.1% filled teeth, 1.2% PUFA and 14.2% toothache, characterizing a population with few dental urgencies

Variables referring to socioeconomic conditions such as age, gender, parent's levels of education and family income were collected.

Training and Calibration

The volunteer's examiners, who are dentists working at Public Service at the municipality, were trained for the caries detection using ICDAS merged codes (Table 1). Examiners were trained and calibrated to evaluate all surfaces of each tooth and classify each one of them according to ICDAS merged codes and ICCMS treatment options codes (Table 2). Also, they were trained to clinical decision making on caries management using ICCMS™.

Table 1. Merged codes ICDAS

CODE	CLASSIFICATION SEVERITY	CHARACTERISTICS OF LESIONS CONNECTED TO SEVERITY
**0**	Sound	No change in enamel in plaque accumulation area
**A**	Initial caries lesion	White spot (translucency other than healthy enamel) or pigmented groove (pigmentation) without loss of surface continuity.
**B**	Moderate caries lesion	Cavitation (or loss of surface continuity) located in opaque or pigmented enamel and / or presence of shading of the underlying dentin.
**C**	Severe caries lesion	Cavitation located in opaque or pigmented enamel with exposure of the underlying dentin.

Table 2. Clinical caries treatment options based ICCMS and adapted for epidemiological study

MERGED CODES -ICDAS	TREATMENT OPTIONS - ICCMS
**A**	**N:** None (only domicile with fluoride dentifrice> 1000 ppmF)
**B**	**TNO:** Non-Operative Treatment (Professional Therapy with Fluorine [Gel or Varnish] and or resin sealants)
**C**	**TO:** Operative Treatment (Restoration with resin, amalgam or restorative ionomer)

One benchmark examiner conducted the training and calibration sessions. Training comprised two training sessions of 4 hours each. The first session included a theoretical explanation and clinical photographic examples. Then, they scored pictures presenting representative scores for both classifications. Subsequently, all the examiners jointly evaluated exfoliated primary teeth and then set in arch models, aided by a dental operating light, 3-in-1 syringe, plane dental mirror and WHO periodontal probe. The teeth and photos used in the calibrations for the diagnosis of caries to represent all caries severity stages. The teeth used were donated by local Human Bank Teeth. One week later, the examinations were repeated to obtain intra and inter-examiner reliability kappa values.

Children's oral examination

The clinical examinations were performed before the child had been vaccinated, in order to prevent manipulation of the oral cavity of the child after receiving the vaccine drops. The clinical examination was conducted on a dental chair using and operating light, a 3-in-1 syringe, plane dental mirror, gauze, WHO ball point probe and individual protection equipment.

Criteria for diagnosis of dental caries (DC)

The child's dental surfaces were cleaned with water-soaked gauze, as recommended by Bönecker et al., 2002 [21], and then the teeth were evaluated wet and then dried with air from the triple syringe.

The criteria to caries lesions assessment was collected according to the ICDAS merged codes for each tooth surface (Table 1). [7] During the collection, the examiners considered the decayed component according to the ICDAS criteria and other components (filled and missing elements) as the proposed by the World Health Organization criteria. [22]

Caries treatment needs

There are five key foundation components of the ICCMS ™, of which are: 1) the staging of the caries process, 2) caries risk classification, 3) the ICCMS ™ decision matrices, 4) ICCMS ™ comprehensive patient management plan, and 5) Outcomes of caries management using ICCMS ™ .[7] Of these keystones, the 1 and 3 were considered. Decision-making was performed after clinical evaluation of the lesion, assuming that all the lesions were active, since that is the reality for most part of caries lesion in this age group.[6] Caries management options were based on merged scores and in accordance to the decision tree described in Table 2.

In the case of moderate lesions, the decision-making adopted was the most conservative, due to the fact that evaluations were performed within an epidemiological study, which there were not radiographic images.

Statistical Analyses

Interexaminer reproducibilities between each trained examiner and the reference examiner were calculated at tooth surface level using a Weighted kappa test for ICDAS merged codes. The intraclass correlation coefficient (ICC) was calculated with 95% of confidence interval (95%CI) considering the absolute agreement among all trained examiners. Thus, systematic differences among examiners were computed.[23]

Caries experience (dmft/dmfs) and caries prevalence were estimated to the studied sample. To assess the impact of including caries lesions at different severity levels in the survey, for all estimates, it was considered 3 thresholds (A- initial, moderate and severe lesions, B- only moderate and severe lesions and C- severe caries lesions). Besides, the prevalence of children presenting restored surfaces were also calculated. dmft/dmfs means values were compared among thresholds and age groups using analysis of variance for repeated measurements. Pairwise comparisons of means of dmft and dmfs were also conducted in order to verify difference among 3 thresholds (A, B and C). To compare prevalence values among thresholds and age groups, the "N-1" Chi-squared test. Bonferroni correction was used for adjustment of significance values in multiple comparisons.

The percentage of children requiring each modality of caries management (none, non-operative care and tooth preserving operative care) was also calculated. Distributions in each modality were compared to other using chi-squared test. Incremental need for non-operative care was also addressed.

Results

After the training on extracted teeth, for staging dental caries, the examiners obtained inter-examiner reproducibility values of weighted kappa agreement between trained examiners and reference examiners varied from 0.62 to 0.80. The ICC (absolute agreement) was 0.65 (95% CI: 0.61 to 0.70), respectively.

664 children and parents/caregivers were invited to participate of the study and a response rate of 99.85% was achieved. Only one child was excluded due to insufficient data collection.

Table 3 shows the socio-demographic characteristics of the sample. The majority of mothers and fathers presented schooling greater than 8 years. About half of the sample presented a family income that corresponded up to two Brazilian minimum wages (1 Brazilian minimum wage = US$224.50). The sample presented a prevalence of 4.1% filled teeth, 1.2% PUFA and 14.2% toothache.

Table 3. Socioeconomic and clinical characteristics of sample (n=663).

**Variables**	**n(%)**
**Child's age (years)**	
2	219 (33.0)
3	225 (34.0)
4	219 (33.0)
**Child's gender**	
Female	326 (49.2)
Male	337 (50.8)
**Mother's levels of education***	
≤8 years	135 (20.4)
> 8 anos	498 (75.1)
**Father's levels of education***	
≤ 8 years	157 (23.7)
> 8 an[illegible]	404 (60.9)
**PUFA**	
Absent	655 (98.8)
Present	8 (1.2)
**Pain**	
Absent	569 (85.8)
Present	94 (14.2)

*n lower than 663 due to missing data

Among children 324 children presented initial caries lesions (49%). On average, each child presented 2 initial caries lesions. The sample presented a prevalence of 4.1% filled teeth, 1.2% PUFA and 14.2% toothache, characterizing a population with few dental urgencies.

The dmft and dmfs increased considerably from cut-off C to cut-off A (Table 4) – p<0.001. Including initial lesions an almost 3-fold increase in dmft was observed (Table 4). when considering initial caries lesions, mean values would be 5x higher compared with case in which the cut-off C was set. The prevalence of caries was increased in cut-off A (inclusion of initial lesions) compared to cut-off B and C (Table 4).This trend was observed in all age groups, but the impact of including initial caries

lesions presented greater magnitude in younger children, especially 2-year-old children.

Table 4. dmfs, dmft and caries prevalence by age range.

		dmfs			dmft			Caries prevalence		
		A	B	C	A	B	C	A	B	C
**Age**	**N**	**Mean (95% CI)**	**Mean (95% CI)**	**Mean (95% CI)**	**Mean (95% CI)**	**Mean (95% CI)**	**Mean (95% CI)**	**% (95% CI)**	**% (95% CI)**	**% (95%IC)**
**2**	219	2.2 aα (1.68-2.75)	0.54 bα (0.30-0.78)	0.39 cα (0.15-0.63)	2.39 aα (1.76-3.01)	0.44 bα (0.27-0.60)	0.24 cα (0.11-0.36)	42.9 aα (0.36-0.49)	15.52 bα (0.11-0.21)	8.6 bα (0.05-0.13)
**3**	225	3.04 aα (2.64-3.78)	1.13 bα (0.73-1.54)	0.77 cα (0.38-1.16)	3.04 aα (2.55-3.53)	0.81bα (0.56-1.07)	0.49 cα (0.26-0.73)	61.3 aπ (0.54-0.67)	28.0 bβ (0.22-0.34)	14.6 cα (0.10-0.20)
**4**	219	5.4 aβ (4.38-6.45)	2.81bβ (2.03-3.59)	2.10 cβ (1.42-2.77)	4.66 aβ (3.83-5.49)	1.81 bβ (1.39-2.22)	1.20 cβ (0.88-1.53)	67.5 aπ (0.60-0.73)	42.0 bπ (0.35-0.48)	31.0 bβ (0.25-0.37)
**Total**	663	3.54 a (3.17-4.05)	1.49 b (1.18-1.80)	1.08 c (0.81-1.36)	3.36 a (2.97-3.75)	1.02 b (0.84-1.19)	0.64 c (0.50- 0.79)	57.3 a (0.53-0.61)	28.5 b (0.25-0.31)	18.1c (0.15-0.21)

A- initial, moderate and extensive stage caries lesions; B- moderate and extensive stage caries lesions; C- extensive stage caries lesions

Different lowercase letters express statistically significant differences within the estimate among the thresholds. Different Greek letters express differences among age groups within the threshold for each estimate. Note: different notations were used to symbolize differences among age groups and thresholds separately, but Bonferroni corrections considered both variables s for adjustments in multiple comparisons.

When analyzing the decision-making observed approximately 40 of children needed NOC and in the 2-year age group more than 50% of the children needed no treatment (N).

Tooth Preserving Operative Care (TPOC) was indicated in minor part of the cases (3%), and more concentrated in older children (Table 6). From 56% of children who required any intervention, most of them (76%) should be essentially scheduled for non-operative care.

Table 6. Distribution of number and percentage of treatment decision by age range

	**ICCMS**				
Age	N	NOC	NOC + TPOC	TPOC	Total
2	126 (57.5)	77 (35)	12 (5.5)	4 (1.9)	219
3	89 (39.5)	108 (48)	22 (9.8)	6 (2.6)	225
4	73 (33)	83 (38)	53 (24.2)	10 (4.5)	219
**Total**	288 (43.4) a	268 (40.4) a	87 (13.2) b	20 (3) c	663

N- none; NOC- Non- Operative Care; TPOC- Tooth Preserving Operative Care

Discussion

Our main results showed that the inclusion of initial caries lesions and decision of caries treatment using ICCMS in the present survey permitted to identify that most part of 2-to-4-year-old children who need treatment in this population would be benefited by NOC. These findings emphasize the importance of using, in some populations, such kind of systems for caries diagnosing, staging and management, differently from those usually employed in conventional epidemiological surveys.

The introduction of noncavitated caries lesions improved sensitivity of caries detection in populations with low prevalence of dental caries, in which caries lesions may present a low progression rate and are found mostly in initial stages.[3] A low caries

experience was observed in the population studied. dmft indices inferior to the Brazilian figures were observed among these children (only franckly cavitated lesions- cut off C: 0.64, microcavitated, undermined and franckly cavitated lesions: 1.02). This low caries experience could be explained by the parents' socio-economic status, since aspects as family income and level of education, have shown association with dental caries.[24] In addition, socioeconomic and environmental indicators, such as the Human Development Index, measures of access to health services and availability of fluoridated water supply, what could influence on dental caries prevalence in certain populations. The municipality of Maua presents Human Development Index of 0.766, which is considered high. It occupies the 131st position in the list of the municipalities of São Paulo and 274th among the Brazilian municipalities.[13]

Initial caries lesions, by themselves, exhibit the higher chance either for reversing or progressing compared to sound surfaces[25] and they show highest chance for change compared to cavitated lesions.[26] That is why they should not be underestimated, being relevant to detect them, when possible. In addition, the initial lesions alone (without being associated with cavitated lesions) were most commonly found in younger children. Probably some of their initial lesions did not have enough time to become cavitated. Caries lesions have a dynamic evolution requiring a long period of time, usually many months or years.[27] In younger children, initial caries lesions have been considered as a predictor of caries progression.

Progression patterns of caries lesions are relevant in the decision-making process[27], since they guide the treatment choices. Mainly, in the initial lesions where the lesions can be managed by non-operative treatment. Therefore, the early diagnosis of these lesions is important for preventive measures to be adopted for their paralysis, and

it is worth emphasizing that when these lesions affect young children the treatment for being more conservative, it allows greater collaboration of the patients.

When the initial stage caries lesions were included, the caries prevalence and caries experience increased, corroborating what has already been described in the literature.[5,6,10] On the other hand, caries detection at this level permitted a group of children, specially the younger ones, could be directed to the non-operative treatments, impacting on demand for treatments. Currently, it has been advocated that the possibility of arresting these lesions would lead to fewer cases in which operative treatment was needed and a lower cost of services could be expected.[28]

A great problem in including initial lesions in epidemiological surveys, especially in developing countries, is planning the actions facing to the reality found. Usually a very high prevalence of cavitated lesions is observed while the initial caries lesions can be detected in almost all children.[5,6] Many Public Health Services are not able to successfully manage this complicated situation in terms of human and financial resources. On the other hand, in such circumstances, the NOC could be extended to all children in need of TPOC, and would be beneficial. Actually, few children in the studied group needed TPOC (operative treatment) and they also did not present many urgencies and treatment priorities, observed by PUFA and pain indices. These findings suggest that for some populations, as that presented in this study, new approaches and strategies should be created to guide the policies to not only to offer restorative treatments, but also treatments that may be focus on those children who present only initial caries lesions, since they are a selected group and population at all.

For this group, summing up 40% of the population, NOC approaches could be planned in public services. The ICCMS™ system provides data for oral health workers

to be able to develop individual preventive strategies and initial stage caries lesions early management, mainly to populations that have many initial caries lesions and little TPOC, such as: motivational interview, diet intervention, prophylaxis, topical fluoride application, oral hygiene guidance with fluoride dentifrice > 1000ppm, glass ionomer and resin sealants. Certainly, we must consider caries activity assessment was not performed in this study, because in epidemiological surveys this criterion exerts little influence on the dental parameters, since most lesions at this age, are active.[6,29]

Even though this protocol may lead to the over-treatment of inactive caries lesions, which would probably represent the minority of lesions. On the other hand, the cost and damage associated with this option, would be minimum since the treatment is non-invasive.

The present study has limitations inherent to the cross-sectional design which does not allow establishing a temporal relationship. However, the present findings highlight the importance of establishing public policies, especially in younger age groups, in order to identify early lesions and treat them, avoiding their progression and consequent cavitation. These orientations could be an additional to modify a panorama of still high prevalence of dental caries at 5-year-old children in some developing countries, as Brazil.[1,17]

Conclusion

The inclusion of initial caries lesions was important in epidemiological survey directed to some populations, because although it significantly increased the caries indicators, it portrayed the greater need of the population, thus, arousing the redirection of the public policies in including Non-Operative Care.

Acknowledgements

The authors thank the local authorities (Health Council), City Hall of the Municipality of Maua, the dental examiners, dental nurses, auxiliary nurses, community agents and the children and their families for their cooperation in carrying out this study. Also, we wish to thank the participants of the Post-Graduate Pediatric Dentistry Seminar of FOUSP for their critical comments.

References

1. Gimenez T, Bispo BA, Souza DP *et al*. Does the Decline in Caries Prevalence of Latin American and Caribbean Children Continue in the New Century? Evidence from Systematic Review with Meta-Analysis. PLoS One. 2016; 11:1-14
2. Pitts N."ICDAS"-an international system for caries detection and assessment being developed to facilitate caries epidemiology, research and appropriate clinical management. Community Dent Health. 2004; 21:193-8.
3. Assaf AV, de Castro Meneghim M, Zanin L, Tengan C, Pereira AC. Effect of different diagnostic thresholds on dental caries calibration – a 12 month evaluation. Community Dent Oral Epidemiol. 2006; 34:213–9.
4. Cadavid AS, Lince CM, Jaramillo MC. Dental caries in the primary dentition of a Colombian population according to the ICDAS criteria. Braz Oral Res. 2010; 24:211–6.

5. Braga MM, Oliveira LB, Bonini GA, Bönecker M, Mendes FM. Feasibility of the International Caries Detection and Assessment System (ICDAS-II) in epidemiological surveys and comparability with standard World Health Organization criteria. Caries Res. 2009; 43:245-9.
6. Piovesan C, Ardenghi TM, Guedes RS, Ekstrand KR, Braga MM, Mendes FM. Activity assessment has little impact on caries parameters reduction in epidemiological surveys with preschool children. Community Dent Oral Epidemiol. 2013; 41:204- 11.
7. Ismail AI, Pitts NB, Tellez M *et al*. The International Caries Classification and Management System (ICCMS™) An Example of a Caries Management Pathway. BMC Oral Health. 2015;15 Suppl 1:S9.
8. Pitts NB, Ekstrand KR. ICDAS Foundation: International Caries Detection and Assessment System (ICDAS) and its International Caries Classification and Management System (ICCMS) – methods for staging of the caries process and enabling dentists to manage caries. Community Dent Oral Epidemiol. 2013; 41:41-52.
9. Pitts NB, Ismail AI, Martignon S, Ekstrand K, Douglas GVA, Longbottom C. ICCMS™ Guide for Practitioners and Educators. Available from: https://www.icdas.org/uploads/ICCMSGuide_Full_Guide_With_Appendices_UK.pdf.2014 Accessed March 19, 2019.
10. Cortes A, Ekstrand KR, Gamboa LF, González L, Martignon S. Caries status in young Colombian children expressed by the ICCMS™ visual/radiographic combined caries staging system. Acta Odontol Scand. 2017; 75:12-20.

11. Martignon S, Cortes A, Gómez SI *et al*. How Long does it Take to Examine Young Children with the Caries ICDAS System and how do they Respond?. Braz Dent J.2018; 29:374-380.
12. Vandenbroucke JP, Von Elm E, Altman DG, *et al*. Strengthening the Reporting of Observational Studies in Epidemiology (STROBE): explanation and elaboration. PLoS Med 2007; 4:e297.
13. SB Brasil 2010. Pesquisa Nacional de Saúde Bucal. Resultados Principais. Brasilia-DF.Edit Ministério da Saúde, 2012. Available from: http://bvsms.saude.gov.br/bvs/publicacoes/pesquisa_nacional_saude_bucal.pdf Accessed March 19, 2019.
14. Roncalli AG. The SB Brasil 2010 Project: a key strategy for developing an oral health surveillance model. Cadernos de saude publica. 2010; 26:428-9.
15. Oliveira LB, Marcenes W, Ardenghi TM, Sheiham A, Bönecker M. Traumatic dental injuries and associated factors among Brazilian preschool children. Dent Traumatol. 2007; 23:76–81.
16. de Vasconcelos Cunha Bonini GA, Marcenes W, Oliveira LB, Sheiham A, Bönecker M. Trends in the prevalence of traumatic dental injuries in Brazilian preschool children. Dent Traumatol. 2009; 25:594–8.
17. Bönecker M, Ardenghi TM, Oliveira LB, Sheiham A, Marcenes W. Trends in dental aries in 1- to 4-year-old children in a Brazilian city between 1997 and 2008. Int J Paediatr Dent. 2010; 20:125–31.
18. Abanto J, Tello G, Bonini GC, Oliveira LB, Murakami C, Bönecker M. Impact of traumatic dental injuries and malocclusions onquality of life of preschool children: a population-based study. Int J Paediatr Dent. 2015; 25: 18–28.

19. Murakami C, Tello G, Abanto J, Oliveira LB, Bonini GC, Bönecker M. Trends in the prevalence of erosive tooth wear in Brazilian preschool children. Int J Paediatr Dent. 2015; 26:60-5.
20. Tello G, Bonini GC, Murakami C, Abanto J, Oliveira LB, Bönecker M. Trends in the prevalence of traumatic crown injuries and associated factors in Brazilian preschool children: 10-year observational data. Dent Traumatol. 2016; 32:274-80.
21. Bönecker M, Marcenes W, Sheiham A. Caries reduction between 1995, 1997 and 1999 in preschool children in Diadema, Brazil. Int J Paediatr Dent. 2002; 12:183-8.
22. World Health Organization. (1997). Oral health surveys: basic methods, 4th ed. World Health Organization. http://www.who.int/iris/handle/10665/41905
23. McGraw KO, Wong SP (1996) Forming inferences about some intraclass correlation coefficients. Psychological Methods. 1996; 1:30-46. (Correction:1:390).
24. Oliveira LB, Sheiham A, Bönecker M. Exploring the association of dental caries with social factors and nutritional status in Brazilian preschool children. Eur J Oral Sci. 2008; 116:37-43.
25. Guedes RS, Piovesan C, Floriano I, *et al*. Risk of initial and moderate caries lesions in primary teeth to progress to dentine cavitation: a 2-year cohort study. Int J Paediatr Dent. 2016; 26:116-24.
26. Pitchika V, Kokel C, Andreeva J *et al*. Longitudinal study of caries progression in 2- and 3-year-old German children. Community Dent Oral. Epidemiol. 2016; 44:354-63.

27. Takahashi N, Nyvad B. Caries ecology revisited: microbial dynamics and the caries process. Caries Res. 2008; 42:409–18.
28. Kidd E, Fejerskov O. Changing concepts in cariology: forty years on. Dental update. 2013; 40:277-8, 280-2, 285-6.
29. Pinto-Sarmento TC, Abreu MH, Gomes MC *et al*. Determinant Factors of Untreated Dental Caries and Lesion Activity in Preschool Children Using I CDAS. PLoS One. 2016; 22.
