# Supplementary material for: Inclusion of initial caries lesions in a population-based sample of Brazilian preschool children: Impact on estimates and treatment needs
Source: PLoS One. 2020 Jun 19;15(6):e0234122. doi: 10.1371/journal.pone.0234122 (PMC7304588; doi:10.1371/journal.pone.0234122)
Supplement: Attachment — Submitted filename: Response to Reviewers.docx [file pone.0234122.s007.docx]

ANSWER LETTER

Dear Reviewers,

we respectfully thank the reviewers for their valuable contributions. Their respective suggestions request for correction that certainly qualify and give greater consistency to our text.

We describe below the treatment given to each of the amendments proposed by the reviewers for re-submission of manuscript PONE-D-19-32314: “Inclusion of initial caries lesions in a population-based sample of Brazilian preschool children: impact on estimates and treatment needs. “ Changes in the revised manuscript were highlighted. “

**METHODS:**

1. Reviewers' comments: It will be good if the reference number for the ethical approval can be included.

Authors' answers:

The ethics and research approval number was included in revised  document (lines 93-94).

2. Reviewers' comments: is this study among five year old or children below 5-year?

Authors' answers:

In fact, the study was carried out on children from 2 to 4 years-old, but the sample size was calculated based on caries prevalence found in a National Brazilian Oral Health Survey carried out by the Ministry of Health that included an age group of   5 years-old Brazilian children (SB Brasil 2010 - Roncalli AG. The SB Brazil 2010 Project: a key strategy for developing an oral health surveillance model. Public health notebooks. 2010; 26: 428-9*). This National Brazilian Survey evaluated individuals in the following age groups: 5yo,12yo, 15-19yo, 35- 44yo and 65-74 years old, according to the WHO criteria.  The revised version also clarifies this point (lines 104-106).

*http://bvsms.saude.gov.br/bvs/publicacoes/pesquisa_nacional_saude_bucal.pdf

3. Reviewers' comments: PUFA is an index I think not percentage

Authors' answers:

We certainly agree with the reviewer and thank him,her for the comment. Actually, the idea was to present the percentage of children who presented any condition assessed in the PUFA index. That is why we rewrote as follows “Only 4.1% of children presented filled teeth, while 14.2% reported any episode of toothache and 1.2% presented pulp polyp, ulcer, fistula or abscess (Monse et al., 2010), characterizing a population with few dental urgencies” to improve the comprehension. Besides, this sentence was moved forward to fit better in the sample description (Results section, lines 229-232).

4. Reviewers' comments: What is the value?

Authors' answers:

We are thankful for your comment. This sentence was really equivocated, and we removed it from the revised manuscript.

**RESULTS**

1. Reviewers' comments: Does this mean the education level of the parents? if so please re-write this sentence?

Authors' answers:

We rewrote the sentence, as suggested (lines 226-227).

2. Reviewers' comments: Please check, is PUFA index or in percentage? the author did not mention PUFA under methodology

Authors' answers:

We certainly agree with the reviewer and thank him,her for the comment. Actually, the idea was to present the percentage of children who presented any condition assessed in the PUFA index. That is why we rewrote as follows “Only 4.1% of children presented filled teeth, while 14.2% reported any episode of toothache and 1.2% presented pulp polyp, ulcer, fistula or abscess (Monse et al., 2010), characterizing a population with few dental urgencies” to improve the comprehension (lines 229-232).

In the methods section (lines 169-71), we included the of the assessment of urgent dental needs and episodes of toothache.

3. Reviewers' comments: What is the meaning of this?

Authors' answers:

This language mistake was corrected in the revised version.

4. Reviewers' comments: percentage should be included.

      Authors' answers:

It was a flawed act and the table will be amended as follows: 219 (33), 225 (34), 219 (33) and 663 (100).

We are at your disposal for any necessary clarifications.

Sincerely,

The Authors
